# Using Breeding Populations With a Dual Purpose: Cultivar Development and Gene Mapping—A Case Study Using Resistance to Common Bacterial Blight in Dry Bean (*Phaseolus vulgaris* L.)

**DOI:** 10.3389/fpls.2021.621097

**Published:** 2021-02-26

**Authors:** Kristin J. Simons, Atena Oladzad, Robin Lamppa, Maniruzzaman, Phillip E. McClean, Juan M. Osorno, Julie S. Pasche

**Affiliations:** ^1^Department of Plant Sciences, North Dakota State University, Fargo, ND, United States; ^2^Department of Plant Pathology, North Dakota State University, Fargo, ND, United States

**Keywords:** GWAS – genome-wide association study, breeding population, common bacterial blight resistance, candidate gene analyses, lipoxygenase, marker assisted breeding, dry bean (*Phaseolus vulgaris* L.)

## Abstract

Dry bean (*Phaseolus vulgaris* L.) is an important worldwide legume crop with low to moderate levels of resistance to common bacterial blight (CBB) caused by *Xanthomonas axonopodis* pv. *phaseoli*. A total of 852 genotypes (cultivars, preliminary and advanced breeding lines) from the North Dakota State University dry bean breeding program were tested for their effectiveness as populations for genome-wide association studies (GWAS) to identify genomic regions associated with resistance to CBB, to exploit the associated markers for marker-assisted breeding (MAB), and to identify candidate genes. The genotypes were evaluated in a growth chamber for disease resistance at both the unifoliate and trifoliate stages. At the unifoliate stage, 35% of genotypes were resistant, while 25% of genotypes were resistant at the trifoliate stage. Libraries generated from each genotype were sequenced using the Illumina platform. After filtering for sequence quality, read depth, and minor allele frequency, 41,998 single-nucleotide polymorphisms (SNPs) and 30,285 SNPs were used in GWAS for the Middle American and Andean gene pools, respectively. One region near the distal end of Pv10 near the SAP6 molecular marker from the Andean gene pool explained 26.7–36.4% of the resistance variation. Three to seven regions from the Middle American gene pool contributed to 25.8–27.7% of the resistance, with the most significant peak also near the SAP6 marker. Six of the eight total regions associated with CBB resistance are likely the physical locations of quantitative trait loci identified from previous genetic studies. The two new locations associated with CBB resistance are located at Pv10:22.91–23.36 and Pv11:52.4. A *lipoxgenase-1* ortholog on Pv10 emerged as a candidate gene for CBB resistance. The state of one SNP on Pv07 was associated with susceptibility. Its subsequent use in MAB would reduce the current number of lines in preliminary and advanced field yield trial by up to 14% and eliminate only susceptible genotypes. These results provide a foundational SNP data set, improve our understanding of CBB resistance in dry bean, and impact resource allocation within breeding programs as breeding populations may be used for dual purposes: cultivar development as well as genetic studies.

## Introduction

Dry bean (*Phaseolus vulgaris* L.) is the most important grain legume directly consumed by humans (Kelly, 2010; Kalavacharla et al., 2011). It provides a primary and/or secondary source of protein, carbohydrates, and micronutrients to millions of people in poor and developing countries. However, both biotic and abiotic stresses significantly reduce potential seed yield, especially in developing countries where chemical control is cost prohibitive. Genetics offers one of the best and environmentally friendly solutions to many of these production problems.

Traditionally, public plant breeders split their resources between cultivar development and basic research into methods and genetics, thus having two plant populations (Fehr, 1987). The cultivar development or breeding population consists of their elite adapted lines, diverse germplasm lines containing traits of interest, and the various generations from the initial cross through cultivar release. The second population or the basic research population primarily consisted of various biparental populations, where quantitative trait loci (QTL) are mapped. These biparental populations provided important trait knowledge and produced markers that could be used for marker-assisted breeding (MAB).

Prior to the advent of massively parallel sequencing, traditional biparental QTL mapping in dry bean using phenotypes, randomly amplified polymorphic DNA markers, and/or simple sequence repeat markers placed genetic factors into wide intervals (Tar’an et al., 2002; Beattie et al., 2003; Blair et al., 2006, 2012; Pérez-Vega et al., 2010; Wright and Kelly, 2011; Checa and Blair, 2012). Linked markers were particularly useful to track the introgression of genetic factors from the resistant parent into adapted genotypes. However, most markers associated with the traits of interest found in these biparental populations were population specific and have been of limited use in other genetic backgrounds (Miklas et al., 2006). Genotyping-by-sequencing (GBS) uses massively parallel sequencing technologies to discover thousands of single-nucleotide polymorphisms (SNPs) for mapping (He et al., 2014). Association mapping using large, diverse populations and dense SNP collections may identify new QTL, confirm previous QTL, and reduce the interval required for candidate gene identification (Hyten et al., 2010; Song et al., 2015).

Dry bean consists of the Middle American and the Andean gene pools that diverged over 100,000 years ago (Mamidi et al., 2013). The Middle American gene pool is divided into four races based on morphology and ecogeographics: Durango, Jalisco, Mesoamerican, and Guatemala (Singh et al., 1991; Beebe et al., 2000; Díaz and Blair, 2006; Blair et al., 2009; Kwak and Gepts, 2009). The Durango and Jalisco races have been subsequently considered to be a single race and contain the pinto, medium red, pink, and great northern market classes cultivated in the United States (Blair et al., 2006; Kwak and Gepts, 2009; Soltani et al., 2017). The navy and black US market classes fall within the Mesoamerican race (Mensack et al., 2010). The Guatemala race is not commonly cultivated in the United States. The Andean gene pool consists of races Nueva Granada, Peru, and Chile. In the United States, race Nueva Granada (kidney and cranberry market classes) is the primarily cultivated race (Mensack et al., 2010).

The combination of the sequencing and assembly of the *P. vulgaris* genome (Schmutz et al., 2014) and the availability of massively parallel sequencing enabled the use of genome-wide association studies (GWAS) in dry bean using GBS-generated SNP data (Cichy et al., 2015; Kamfwa et al., 2015; Moghaddam et al., 2016; Zuiderveen et al., 2016; Soltani et al., 2017; Oladzad et al., 2019a,b; Zitnick-Anderson et al., 2020). In dry bean, the Middle American and Andean diversity panels were established by individually selecting primarily cultivated genotypes from the two gene pools (Cichy et al., 2015; Moghaddam et al., 2016). Both panels have been used to map various agronomic, food quality, and disease resistance traits (Cichy et al., 2015; Kamfwa et al., 2015; Moghaddam et al., 2016; Zuiderveen et al., 2016; Soltani et al., 2017; Oladzad et al., 2019b; Zitnick-Anderson et al., 2020) with GWAS. These diversity panels are a great tool for mapping genes and finding new sources of genetic variability to incorporate into breeding material.

Studies utilizing highly diverse (and sometimes unadapted) germplasm panels as mapping populations will not identify important QTL found within an existing breeding program. Therefore, using advanced-generation genotypes selected during cultivar development as an association population for genetic mapping will identify SNPs relevant to the breeding program which can be converted to easily distinguishable markers in a MAB program. Toward this goal, Shi et al. (2011) used an average of seven markers per chromosome to look for associations with common bacterial blight (CBB) resistance. Shi et al. (2011) successfully identified 14 of 75 SNP associated with CBB resistance among 465 dry bean breeding genotypes. The use of an average of seven markers per chromosome limited the utility of the markers for MAB or fine-mapping as they may or may not be located close to the resistance genes. Agarwal (2014) improved on the study by Shi et al. (2011) by drastically increasing the number of markers used for association mapping. They used 3,046 markers on 208 breeding lines in the Middle American gene pool. Between nine and 13 highly significant markers were found to be associated with yield, seed weight, plant height, and maturity among the markers evaluated in advanced and preliminary genotypes screened in field trials. In Agarwal’s study, any minor alleles present in less than 5% of their population were eliminated, thereby eliminating rare alleles. With the advancement of sequencing technology and the decrease in sequence cost, hundreds of thousands of SNP can now be identified, and more individuals can undergo sequencing. These studies demonstrate that dry bean breeding populations can be a useful alternative to diversity panels for GWAS, as enough phenotypic diversity exists to provide meaningful associations. The inclusion of tens of thousands of markers and more individuals would allow finer mapping of associated QTL and markers directly applicable to the breeding program.

Common bacterial blight is one of the most important factors limiting dry bean production worldwide (Tar’an et al., 2001; Duncan et al., 2011; Viteri and Singh, 2014). *Xanthomonas axonopodis* pv. *phaseoli* (Smith, 1897) (*Xap*), the causal agent of CBB, causes necrotic lesions sometimes encircled by chlorosis as well as wilting and rot with severe infection (Saettler, 1989; Harveson and Schwartz, 2007; Karavina et al., 2011). The bacterium is seed-transmitted and can overwinter on infected plant debris. *Xap* is disseminated to healthy plants from infected plants by wind, wind-driven rain, irrigation water, hail, people, or machinery (Harveson and Schwartz, 2007; Duncan et al., 2012). Up to 50% yield loss may occur in a conducive environment (Harveson and Schwartz, 2007; Viteri and Singh, 2014). CBB is difficult to control once infection occurs, and an integrated approach for management is required. Crop rotation and planting disease-free seed are important measures to prevent CBB infection. However, genetic resistance is the most effective method of managing CBB.

Common bacterial blight resistance is inherited as both minor- and major-effect QTL and is highly variable depending on disease pressure, environmental conditions, genetic background, and plant maturity (Kelly et al., 2003; Miklas et al., 2006; Durham et al., 2013). Direct screening for resistance is time consuming and labor intensive. MAB can significantly reduce the number of lines and subsequently the time and labor necessary to screen for resistance (Yu et al., 2008). Substantial efforts have been put forth to identify CBB resistance in dry bean, leading to the description of over 25 QTL found across all 11 chromosomes [reviewed by Miklas et al. (2006) and Viteri et al. (2014b)]. Three markers, SU91, BC420, and SAP6, have been used in selecting genotypes with resistance to CBB and have improved the level of CBB resistance present in current cultivars; however, no commercial cultivars are completely resistant to CBB (Yu et al., 2000, 2008; Miklas et al., 2006; O’Boyle et al., 2007; Fourie et al., 2011).

The objective of this study was to determine if lines across various stages of the breeding program could serve a dual purpose for both cultivar development and GWAS, thereby not dividing limited resources between selection/cultivar development and genetic mapping. The CBB resistance phenotype was used as a test case for both the Andean and Middle American gene pools since both major and minor QTLs have been previously described.

## Materials and Methods

### Plant Materials

A total of 852 genotypes in either preliminary or advanced stages in the NDSU dry bean breeding program^1^ were selected for genotyping and CBB phenotyping under controlled conditions in a growth chamber. These genotypes included 32 cultivars, 137 genotypes at the advanced stage, and 683 genotypes at the preliminary stage (Table 1). The genotypes were from nine market classes: dark red kidney, light red kidney, white kidney, great northern, pinto, red, pink, black, and navy. The Andean gene pool population consisted of 139 genotypes, and 713 genotypes belonged to the Middle American gene pool.

**TABLE 1:** Gene pools, races, and market classes of cultivars and North Dakota State University advanced and preliminary breeding genotypes screened for resistance to common bacterial blight under greenhouse conditions and utilized for gene mapping.

	Cultivar	Advanced	Preliminary	Total
**Durango–Jalisco**	16	71	344	431
Great northern	6	19	78	103
Pink	1	4	24	29
Pinto	5	31	170	206
Red	4	17	72	93
**Mesoamerican**	4	62	216	282
Black	4	42	140	186
Navy	0	20	76	96
**Middle American total**	20	133	560	713
**Nueva Granada**	9	4	126	139
Dark red kidney	4	0	61	65
Light red kidney	4	4	45	53
White kidney	1	0	20	21
**Andean total**	9	4	126	139

### Evaluating Resistance to *Xanthomonas axonopodis* pv. *phaseoli*

A highly virulent bacterial isolate of *Xap* collected in Minnesota, Xap f91-5, was used to determine the level of resistance to CBB. Plants were grown in a large growth chamber at 26°C under a 16-h photoperiod with 70% humidity. Bacterial inoculum was prepared from 2- to 3-day-old cultures and diluted to ∼1 × 10^7^ colony-forming units/ml (Mutlu et al., 2008; Fourie and Herselman, 2011; Miklas et al., 2011; Duncan et al., 2012) in 12.5 mM potassium phosphate buffer (pH 7.1; Miklas et al., 1996; Mutlu et al., 2008). Each selected leaf was inoculated on either side of the abaxial vein at 10 (unifoliate) and 21 days (trifoliate) post-planting using an air brush sprayer (Singh and Muñoz, 1999; O’Boyle et al., 2007; Tryphone et al., 2012). Disease reactions for each leaf were evaluated 14 days post-inoculation using a 1-to-9 scale (Aggour et al., 1989), where 1 is no visible reaction, and 9 is 91% of the leaf area is exhibiting chlorosis and/or necrosis. The experiment consisted of an augmented design with a total of six incomplete blocks planted over 6 months. Each block consisted of three replicates of 100–167 test genotypes plus two check genotypes, the germplasm line ‘Vax 3’ (Singh et al., 2001) and the cultivar ‘Montcalm’. Each replicate consisted of one plant per genotype with two inoculated unifoliate leaves and two inoculated trifoliate leaves. PROC GLM in SAS 9.4 (SAS Institute, Cary, NC, United States) was used to analyze the infection variation among the repeating check genotypes. No significant differences were found in the rating of the check genotypes throughout the trial, indicating a uniformity of infection. Thus, no block adjustment was conducted on the ratings, and subsequently the data from all blocks was combined. Genotypes with a median of three or below for each trait were considered as resistant. Descriptive statistics were estimated for all traits using JMP Pro 15 (SAS Institute, Cary, NC, United States).

### DNA Extraction

Approximately 50 mg of young trifoliate leaf tissue was harvested from a single inoculated plant of each genotype grown in the growth chamber, and the DNA was extracted using the Mag-Bind Plant DNA Plus Kit (Omega bio-tek, Norcross, GA, United States) using the KingFisher Flex (ThermoScientific) for bead washing. DNA was quantified using a nanodrop, diluted to 50 ng/μl, and checked for quality *via* gel electrophoresis.

### SNP Data Set

The breeding line SNP data set was obtained by low-pass sequencing of libraries generated using a two-enzyme protocol (*TaqI* and *MseI*; Moghaddam et al., 2016; Schröder et al., 2016). Each sequencing library consisted of 95 uniquely barcoded samples and was sequenced by the HudsonAlpha Institute of Biotechnology, Huntsville, AL, United States, using a single lane of an Illumina Hi-Seq in rapid run mode.

The sequencing quality of each run was verified with FASTQC v0.11.5 (Andrews, 2010). Each pool of 200-bp reads was decomposed into individual breeding lines based on barcode using FASTX v0.0.14^2^. Genotyping was repeated for any individual that generated less than 100,000 reads. FASTX was used to trim the barcode sequences from the reads. The reads underwent quality trimming using SICKLE (Joshi and Fass, 2011) when the Phred score was less than 20 and discarded if less than 80 bp remained after quality trimming. BWA-MEM (Li and Durbin, 2009; Li, 2013) and SAMtools (Li et al., 2009) were used to align, index, and sort the reads against the reference genome (*Phaseolus vulgaris* v2.1, DOE-JGI and USDA-NIFA^3^). Read groups including library ID, platform, and platform unit were added to each alignment within the BAM files using Picard^4^. Alignments for each breeding line were divided to generate gene pool-specific SNP data sets. Each gene pool has undergone separate domestication bottlenecks and selection pressure; thus, the SNP data sets were generated separately (Mamidi et al., 2013; Schmutz et al., 2014; Moghaddam et al., 2016; Soltani et al., 2017; Oladzad et al., 2019a,b). If combined, the SNP data set would be very large and contain a large number of SNP that are gene pool specific. The target variants for identification were biallelic SNPs; therefore, unified genotyper from GATK3.6 (DePristo et al., 2011) was used to call variants with quality scores above 10. Quality scores between 10 and 30 were marked as low quality. Variants with a read depth of less than two were filtered using GATK3.6 variant filtration and subsequently replaced as missing data. Low-quality variants were removed *via* hard filtering when variants contained more than 25% missing data (50% in the MA SNP data set), more than one nucleotide, and more than two alleles or the minor allele was less than 5% (<1% in the MA SNP data set). The minor allele frequency (MAF) was reduced to less than 5% to allow the detection of rare alleles in the breeding population. The MAF was set to the equivalent of five individuals in each population as the presence of the allele in five genotypes suggests the presence of a true allele. The data set may be filtered to remove additional SNP with MAF below 5% for future use. Genotypes with more than 90% missing data were removed. SNPs with less than 25% (50% in MA SNP data set) of missing data were imputed in fastPHASE (Scheet and Stephens, 2006). The SNP data sets were deposited in the NDSU repository (Simons et al., 2020).

### Genome-Wide Association Studies

Genome-wide association studies analyses were performed for both the Andean and Middle American gene pools. GEMMA (Zhou and Stephens, 2012, 2014) was used to complete the GWAS analyses on both trait data as it is capable of testing phenotypes regardless of the underlying distribution. The principal component analysis (PCA) was completed using the R3.5 function prcomp() on each SNP data set (Price et al., 2006; R Core Team, 2019). The number of PCAs that accounted for 20–50% of the variation-identified associations for each trait was included as a fixed effect in the model. A generalized linear mixed model using the center-relatedness algorithm as a random effect within GEMMA identified associations for each trait. A multivariate mixed model using both phenotypes was used to complement the univariate results, as any genomic region with significance in both types of analysis is more likely to be true and not a type I error. To determine if the effect size was significantly different than zero, a Wald test in GEMMA was performed. The mhtplot() function within the R3.5 package gap (Zhao, 2007) was used to generate the final Manhattan plots. Significant *p*-values were determined and set at the lowest 0.01 and 0.1% of the empirical distribution of *p*-values after 1,000 bootstraps were considered (Mamidi et al., 2014; Moghaddam et al., 2016; Oladzad et al., 2019a,b, 2020) and drawn as horizontal bars on the Manhattan plots. As referenced by Mamidi et al. (2014) and Oladzad et al. (2020), artificial cutoffs such as those from the Bonferroni test work best for traits in which only a few genetic factors are involved. For other traits, a better way to define the cutoff values is based on the trait and population. Bootstrapping with 1,000 resamplings “is sensitive to the fact that the more genetic factors affecting a phenotype, the corresponding values of *p* would be higher” (Oladzad et al., 2020). CBB resistance is a complex trait with many genetic factors affecting resistance, and thus bootstrapping with resampling provides a more applicable set of cutoff values. The amount of variation explained by the significant SNPs was calculated in the genABEL R package using a likelihood-ratio-based R2 (R2LR) analysis (Sun et al., 2010). Significant differences between the phenotype averages for each peak SNP were determined using a one-way ANOVA (JMP Pro 14.0, SAS Institute Inc., Cary, NC, United States, 1989–2019).

### QTL Comparison

The physical location of 11 polymerase chain reaction-based markers previously associated with CBB resistance QTL on chromosomes identified as associated with CBB resistance in these GWAS was located on the *Phaseolus vulgaris* v2.1 sequence (Yu et al., 2000, 2008; Tar’an et al., 2001; Miklas et al., 2006; Viteri et al., 2014a). Legume Information System^5^ was searched using the marker name and the corresponding physical location determined. If the marker was not present in the database, primer sequences were input into BLASTN on Phytozome 12^6^ and aligned using the default matrix. A positive match required an *E*-value less than 1.00 × E-30. Any significant SNPs identified with GEMMA were compared to previously reported QTL locations. Candidate genes were identified within 100 kb upstream and downstream of the physical location (Mb) of the peak SNP.

## Results

### Phenotyping

Common bacterial blight reactions can be variable even under greenhouse conditions; therefore, the ratings for the GWAS were conducted in a growth chamber where the conditions were tightly controlled. Under these tightly controlled conditions, the ratings on the control genotypes from each incomplete block were not statistically different. The data for all lines was subsequently combined, and the medians were determined. Median genotype scores ranged from highly resistant (1) to highly susceptible (8) under growth chamber conditions using a highly virulent *Xap* isolate from Minnesota, United States. The median scores for both unifoliate and trifoliate resistance were normally distributed. At the unifoliate stage, 35% of genotypes were resistant, 55% intermediate, and 10% susceptible. At the trifoliate stage, 25% of genotypes were resistant, 69% intermediate, and 6% susceptible. Resistance was not always consistent across growth stages (Table 2 and Supplementary Table 1). Approximately 18% of lines were resistant at both growth stages, while an additional 17% were resistant at the unifoliate stage only, and just under 7% were resistant only at the trifoliate stage. In total, the 139 genotypes that exhibited resistance at both the unifoliate and trifoliate stage can be exploited as genetic sources for CBB resistance during cultivar development. The pinto, great northern, red, and pink market classes had 21, 13, 47, and three genotypes (Middle American gene pool) exhibiting resistance at both growth stages, respectively. Resistance at the unifoliate and trifoliate growth stages was found in only two navy and six black bean genotypes (Middle American gene pool). Among kidney bean genotypes (Andean gene pool), 25 dark red kidney, 16 light red kidney, and six white kidney genotypes exhibited both unifoliate and trifoliate resistance.

**TABLE 2:** Common bacterial blight (CBB) reaction of North Dakota State University advanced and preliminary breeding germplasm evaluated under greenhouse conditions (genotypes missing either a trifoliate or unifoliate rating were removed from this comparison).

Market class	CBB resistance				Total
	RR	RS	SR	SS	
Great northern	13	3	8	72	96
Pink	3	6	0	18	27
Pinto	21	17	20	114	172
Red	47	30	3	7	87
Black	6	40	9	120	175
Navy	2	3	0	79	84
Dark red kidney	25	21	0	16	62
Light red kidney	16	8	8	18	50
White kidney	6	4	3	7	20
Grand total	139	132	51	451	773

*RR, unifoliate and trifoliate resistance; RS, unifoliate resistance and trifoliate susceptibility; SR, unifoliate susceptibility and trifoliate resistance; SS, unifoliate and trifoliate susceptibility.*

### SNP Data Sets

The number of sequencing reads obtained from each breeding genotype ranged from 118,208 to over 11 million. The total number of variants in the MA data set before filtering ranged from 210,000 variants on Pv06 to over 420,000 variants on Pv08. After filtering and combining the SNP data sets from each chromosome, 41,998 SNP variants were discovered for the Middle American population. Pv09 had the fewest number of SNPs with 1,467, and Pv08 had the most with 6,006. Only SNPs with less than 50% missing data underwent imputation. Of those SNPs undergoing imputation, 58% were found in 80% of the genotypes.

The initial count of sequence variants in the Andean population was over 1.3 million SNPs. After filtering for read depth, MAF, and missing data, 30,285 SNPs remained in the data set. The SNPs were dispersed across all 11 chromosomes ranging from 1,007 on Pv10 in the Andean population to 7,075 on Pv11, indicating that little genetic diversity was found on Pv10 and a much higher level on Pv11. The marker density was higher in the Middle American data set with one SNP marker per 14.4 kb when compared with the Andean data set at one SNP marker per 24.8 kb. In the Andean population, only SNPs with less than 25% missing data underwent imputation. Of the SNPs undergoing imputation, 54% were found in 80% of the genotypes.

### Population Structure and Individual Relatedness

**FIGURE 1:**
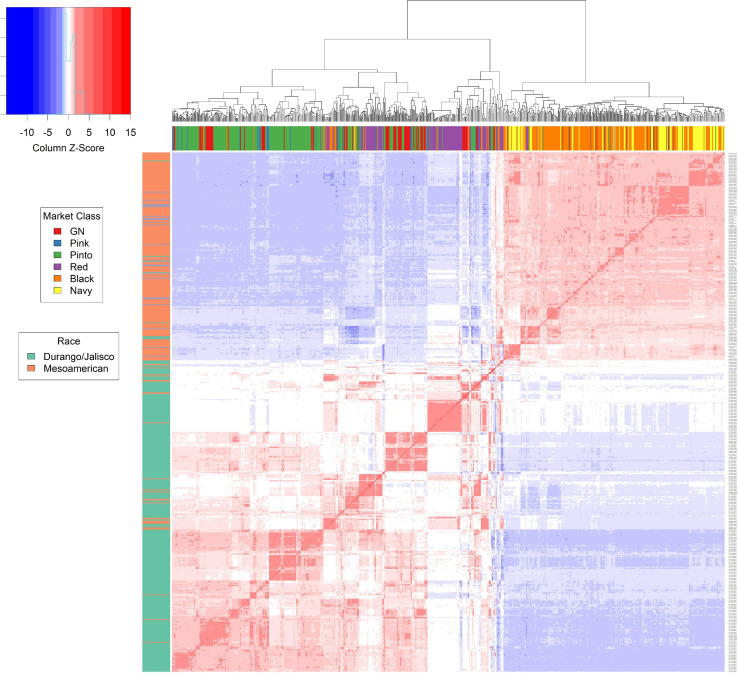
The kinship matrix from GEMMA provides an in-depth view of the relation between each genotype within the Middle American population. The matrix is represented by a heat map. The box plot to left of the heat map represents the race of the genotype based on breeding characteristics. The box plot directly above the heat map displays the market class of each genotype. Hierarchical clustering of the genotypes is depicted above the market class box plot.

The relationship matrix was calculated using the center-relatedness algorithm in GEMMA, and a heat map was generated (Figure 1). The breeding lines in the Middle American gene pool are separated into the Mesoamerican race and the Durango/Jalisco race, as expected, based on earlier reports (Kwak and Gepts, 2009). Admixture existed among market classes within both the Mesoamerican and Durango/Jalisco races (Figure 1). This is likely the result of frequent crosses made between genotypes within the same race (Vandemark et al., 2014). All Andean genotypes, from market classes dark red kidney, light red kidney, and white kidney, exhibited admixture among market classes. The PCA plots were examined, and a principal component (PC) of two was selected to explain 45% of the variation for the Andean population and a PC of one for the Middle American population to explain 22% of the total variation.

### Association Mapping in the Middle American Breeding Population

**FIGURE 2:**
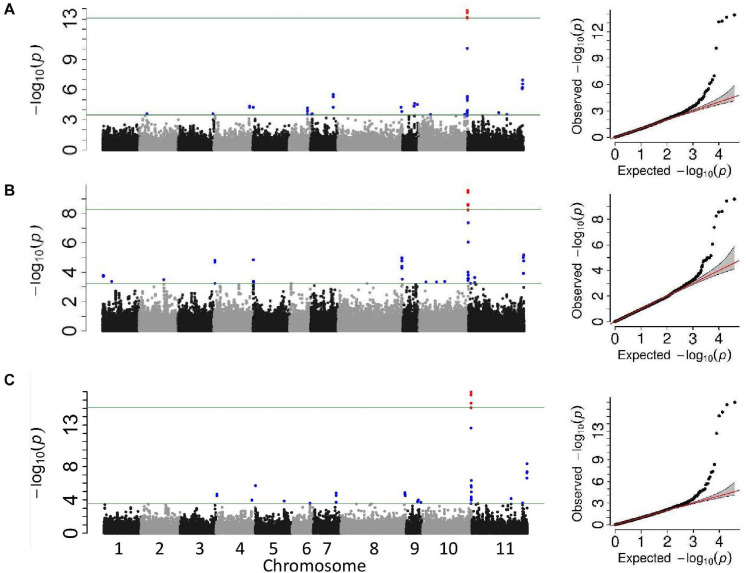
Manhattan and QQ plots from the genome-wide association studies analyses of the Middle American breeding population. The upper horizontal bar represents the cutoff for the most 0.01% significant single-nucleotide polymorphisms (SNP) (depicted in red). The lower horizontal bar represents the cutoff for the 0.1% significant SNP (depicted in blue). Both cutoffs are based on 1,000 bootstraps of the empirical distribution of the *p*-values. **(A)** Univariate mixed model of the unifoliate phenotype, **(B)** univariate mixed model of the trifoliate phenotype, and **(C)** multi-variate mixed model of unifoliate and trifoliate phenotype.

Two GWAS analyses, for unifoliate and trifoliate resistance, using a univariate mixed model in GEMMA detected eight significant linkage blocks in the Middle American breeding population (Figures 2A,B), one unique to the unifoliate, five unique to the trifoliate, and two in common. The location, Pv10:41.66–41.84, contained highly significant SNPs in both analyses. It explained 16.9% of the total variation within the unifoliate phenotype and 10.8% in the trifoliate phenotype (Table 3). The peak SNP, S10_41,784,824, is found within a cluster of 13 *lipoxygenase* (LOX) 1-like genes. It lies 11 kb downstream of gene model *Phvul.010G135151*, a LOX 1-like gene, and 246 bp upstream of gene model *Phvul.010G135200*, which putatively encodes a protein belonging to the calcium-binding endonuclease/exonuclease/phosphatase family. Besides the location on Pv10, two additional locations with significant SNPs were identified at Pv07:28.50–28.84 and Pv11:52.40 (Table 3) in the unifoliate analysis. The peak SNPs for each of these two locations explained 7.5 and 7.4% of the total variation, respectively. The three physical locations combined explain 27.7% of the cumulative variation in the unifoliate phenotype. The physical location around the peak SNP, S07_28,772,508, encodes a number of proteins previously implicated in disease resistance, including a glutathione peroxidase, a tetratricopeptide repeat (TPR)-like protein, a BTB-POZ and MATH domain protein, a pentatricopeptide repeat (PPR) protein, and a receptor-like protein kinase-related family protein (Boyle et al., 2009; Kwon et al., 2009; Laluk et al., 2011; Park et al., 2014). The Pv11:52.40 location contains two TPR-like proteins as well as glycoside hydrolase, both of which are implicated in disease resistance. The peak SNP, S11_52,401,283, is located 44 kb downstream of gene model *Phvul.011G207000*, a TPR-like superfamily protein, and 15 kb downstream of *Phvul.011G206800*, a glycoside hydrolase family 2 protein.

**TABLE 3:** GEMMA GWAS results for phenotypic traits in the Middle American breeding population.

Phenotypic data	Type of analysis	Location		Peak SNP			
		Chromosome	Genomic	Position	–log10	Variation	Cumulative
			location (Mb)	(Mb)	(*p*)	(%)	variation (%)
Unifoliate	Univariate	7	28.50–28.84	S07_28,772,508	5.5	7.5	27.7
		10	41.66–41.84	S10_41,784,824	13.9	16.9	
		11	52.4	S11_52,401,283	7.0	7.4	
Trifoliate	Univariate	4	1.52	S04_1,523,805	4.7	5.2	25.8
		5	0.17–0.19	S05_178,530	4.8	5.2	
		8	62.39–62.40	S08_62,399,301	5.0	5.8	
		10	22.91–23.36	S10_23,164,707	4.9	6.4	
		10	41.66–41.84	S10_41,784,824	9.4	10.8	
		10	42.26–42.52	S10_42,412,927	7.4	8.5	
		11	52.4	S11_52,401,283	5.2	5.7	
Multi-trait	Multivariate	5	0.17–0.19	S05_178,530	5.7		
		7	28.50–28.84	S07_28,772,508	4.8		
		8	62.39–62.40	S08_62,399,327	4.9		
		10	41.66–41.84	S10_41,784,824	17.0		
		10	42.26–42.52	S10_42,412,927	6.3		
		11	52.4	S11_52,401,283	8.3		

The trifoliate GWAS identified a total of seven physical locations associated with CBB resistance. The Pv10:41.66–41.84 and Pv11:52.40 locations are common between both the unifoliate and trifoliate phenotypes (Table 3). In addition to these two locations, five significant locations located on Pv04, Pv05, Pv08, and Pv10 were associated with the trifoliate phenotype. The peak SNP in the Pv04:1.52 location, S04_1,523,805, is downstream from a cluster of NB-ARC domain-containing disease resistance proteins located at Pv04:1.43–1.51. The physical location around S05_178,530 encodes a number of proteins. It is 24 kb upstream of a cytochrome 450 protein and 29 kb downstream of a phosphatidylinositol-specific phospholipase C protein, both of which play a role in disease resistance (reviewed in Xu et al., 2015; Abd-El-Haliem and Joosten, 2017). Within location Pv08:62.40, S08_62,399,301 is the peak SNP and is located 224 bp upstream of gene model *Phvul.008G283900*, a proliferating cell nuclear antigen 2 ortholog. Few genes were found within 100 kb of S10_23,164,707; however, it is between a pair of leucine-rich repeat receptor-like protein kinases found 175 kb proximal and 139 kb distal to the peak SNP. Leucine-rich repeat receptor protein kinases play a vital role in plant defense (reviewed in Belkhadir et al., 2014). The location containing SNP S10_42,412,927 encompasses multiple encoded proteins, including a cluster of pyridoxal phosphate phosphatase-related proteins which have been implicated in *Pseudomonas* disease severity and the development of the hypersensitive response (Denslow et al., 2005). The seven peak SNPs for these locations explained 25.8% of the variation in the trifoliate phenotype.

Multivariate mixed model analyses provided further evidence for six of the CBB resistance-associated locations identified in the two univariate mixed model analyses (Figure 2C). Two locations, Pv10:41.66–41.84 and Pv11:52.40, were found in all three analyses (Table 3), with the location on Pv10 containing the most significant SNP, S10_41,784,824 (–log10 *p* = 17.0). The locations on Pv05, Pv07, Pv08, and Pv10 identified in the univariate analysis were confirmed in the multivariate analysis.

The one-way ANOVA analyses for each nucleotide state indicated that the means for the two homozygous states were statistically different for each of the six significant SNPs confirmed by the multivariate analysis (Table 4). The lack of significant differences between the heterozygous state and the homozygous state for five of the six significant SNPs suggests dominant/recessive gene action. The mean of heterozygous nucleotide state for SNP S10_41,784,824 is significantly different from the means of both homozygous alleles, which provides preliminary evidence of a different mode of action, such as incomplete dominance.

**TABLE 4:** Relationship between the allelic state of peak single-nucleotide polymorphisms (SNPs) and their utility in marker-assisted breeding in the Middle American breeding population for SNPs identified by at least two of the three analyses.

SNP		Disease rating^a^				Number of genotypes in combined phenotype^b^				
	SNP nucleotide	Unifoliate		Trifoliate		RR	RS	SR	SS	Total
S05_178,530										
	CC	2.5	A	3.2	A	3	2			5
	GC	2.5	A	3.2	A	47	23	4	4	78
	GG	4.7	B	4.6	B	43	76	36	416	571
S07_28,772,508										
	CC	4.2	A	4.4	A	91	97	38	328	554
	TC	4.6	A	4.1	AB	2	1	1	4	8
	TT	5.9	B	5.1	B		3	1	88	92
S08_62,399,301										
	AA	3.1	A	3.6	A	6	5	1	3	15
	TA	3.1	A	3.4	A	42	22	10	16	90
	TT	4.7	B	4.7	B	45	74	29	401	549
S10_41,784,824										
	AA	3.5	A	3.9	A	70	51	12	89	222
	AG	4.1	B	4.3	B	11	16	3	31	61
	GG	5.1	C	4.8	C	12	34	25	300	371
S10_42,412,927										
	CC	5.0	A	4.8	A	15	40	17	263	335
	CT	3.5	B	4.3	B	10	13	1	16	40
	TT	3.9	B	4.1	B	68	48	22	141	279
S11_52,401,283										
	AA	4.7	A	4.7	A	43	73	36	416	568
	AG	2.6	B	3.3	B	36	23	4	4	67
	GG	2.3	B	3.0	B	14	5			19

*RR, unifoliate and trifoliate resistance; RS, unifoliate resistance and trifoliate susceptibility; SR, unifoliate susceptibility and trifoliate resistance; SS, unifoliate and trifoliate susceptibility. ^*a*^Different letters within columns represent significant differences between phenotypes based on Tukey–Kramer’s analysis. ^*b*^Genotypes missing either the unifoliate or trifoliate score were removed from this analysis.*

### Association Mapping in the Andean Breeding Population

**FIGURE 3:**
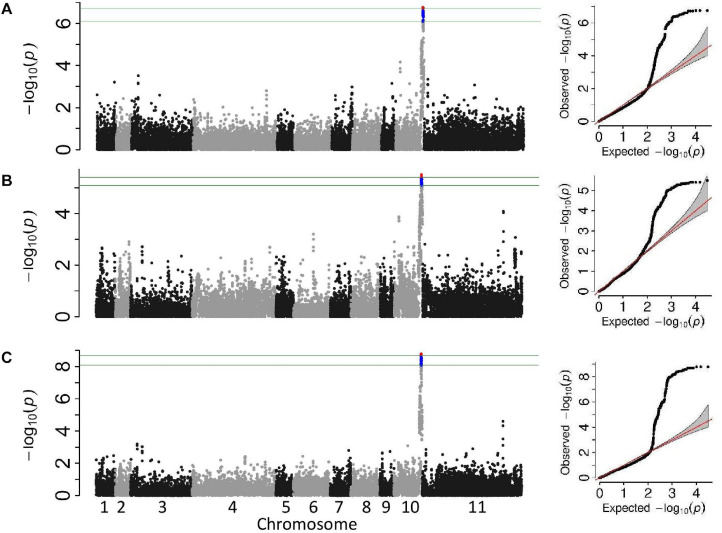
Manhattan and QQ plots from the genome-wide association studies analyses of the Andean breeding population. The upper horizontal bar represents the cutoff for the most 0.01% significant single-nucleotide polymorphisms (SNP) (depicted in red). The lower horizontal bar represents the cutoff for the 0.1% significant SNP (depicted in blue). Both cutoffs are based on 1,000 bootstraps of the empirical distribution of the *p*-values. **(A)** Univariate mixed model of the unifoliate phenotype, **(B)** univariate mixed model of the trifoliate phenotype, and **(C)** multi-variate mixed model of unifoliate and trifoliate phenotype.

Two association analyses, unifoliate and trifoliate, were performed on the phenotypic data using a univariate mixed model. Both analyses identified a single significant genomic location on Pv10 (Figures 3A,B). The significant genomic location consisted of all SNPs adjacent to the significant peak SNP with similar MAF. The peak SNPs from both analyses were within the location Pv10:41.11–42.22Mb (Table 5). The most significant SNP within this linkage block explained 34.2% of the variation within the unifoliate phenotype and 26.7% in the trifoliate phenotype (Table 5). The peak SNPs in this cluster are bordered downstream by a TIR-NBS-LRR disease resistance cluster and are downstream of a LOX 1-like cluster.

**TABLE 5:** GEMMA GWAS results for phenotypic traits in the Andean breeding population.

Phenotypic data	Type of Analysis	Location		Peak SNP		
		Chromosome	Genomic location (Mb)	Position (Mb)	–log10 (*p*)	Variation (%)
Unifoliate	Univariate	10	41.11–42.22	S10_41,522,006	6.8	34.2
Trifoliate	Univariate	10	41.11–42.22	S10_41,645,518	5.5	26.7
Multi-trait	Multivariate	10	41.11–42.22	S10_41,645,484	8.8	

A single significant peak was obtained from the multivariate mixed model using both the unifoliate and trifoliate phenotypic data (Figure 3C). The most significant SNP, S10_41,645,484 (–log10 *p* = 8.8), was 120 kb distal of the most significant SNP found in the unifoliate analysis and 34 bp proximal of the most significant SNP found in the trifoliate analysis (Table 5). The similar location obtained from the multivariate analysis further validates the results from the univariate analysis.

### Comparison to Previously Reported QTL

Over 25 QTL associated with CBB resistance have been identified in previously published research (reviewed in Miklas et al., 2006; Viteri et al., 2014a). The GWAS analyses performed here identified eight locations that contributed to CBB resistance within the NDSU dry bean breeding program. Six of these locations are near previously identified QTL (Table 6). The only significant location from the Andean GWAS, Pv10:41.11–42.22 Mb, is near the SAP6 marker (41.0 Mb) used for MAB (Miklas et al., 1996, 2000). Marker SNP47812 was associated with the resistance QTL near SAP6 but at 41.54 MB (Viteri et al., 2014a). The Middle American GWAS identified seven additional locations associated with CBB resistance, which were compared to the locations of markers near CBB QTL from the composite map (Miklas et al., 2006). SU91, located at 62.84 Mb, is near the CBB resistance location Pv08:62.39–62.40 identified in this study. The location Pv05:0.16–0.19 Mb, near the end of Pv05, may be the same CBB QTL associated with marker G19.1800, which has no physical location but has been placed at the end of Pv05. The location Pv07:28.50–28.84 may overlap a previously identified CBB QTL near marker bng204, located at 25.71 Mb. Four CBB QTL were identified on Pv11, three from the consensus map (Miklas et al., 2006) and one from Viteri et al. (2014a). Viteri et al. (2014a) identified a location, Pv11:44.90–45.63, near the end of the chromosome associated with CBB resistance. The location, Pv11:52.4, identified in this study does not overlap with any known locations and is a new location associated with CBB resistance. No QTL has been identified near the Pv10:22.91–23.36 location, and therefore, this represents a second newly associated location with CBB resistance identified in this study.

**TABLE 6:** Comparison of the physical locations of markers previously associated with common bacterial blight (CBB) resistance similar to the locations identified in the NDSU breeding program.

SNPs associated with CBB resistance			Previously published QTL/marker		
SNP	Chromosome	Location	Marker	Chromosome	Location
S04_1,523,805	4	1.52	bng071^a^	4	5.02
			Pv-CTT001^b^	4	0.52
S05_178,530	5	0.17–0.19	bng162^a^	5	40.45
			G19.1800^a^	5	Unknown
S07_28,772,508	7	28.50–28.84	BC420^*c^	7	17.33
			bng204^d^	7	25.71
S08_62,399,327	8	62.39–62.40	bng073^d^	8	61.90
			SU91^*e^	8	62.84
S10_23,164,707	10	22.91–23.36	SAP6^*e^	10	41.05
S10_41,784,824	10	41.66–41.84	SNP47812^f^	10	41.54
S10_42,412,927	10	42.26–42.52			
S11_52,401,283	11	52.4	SNP47588^f^	11	44.90-45.63

*Markers previously used in marker-assisted breeding for CBB resistance. ^a^Tar’an et al., 2001. ^b^Yu et al., 2008. ^c^Yu et al., 2000. ^d^Miklas et al., 2006. ^e^Miklas et al., 2000. ^f^Viteri et al., 2014a.

## Discussion

Association mapping using breeding germplasm identifies genetic factors controlling traits important to regions targeted by the breeding program. The results can be used to identify SNPs associated with favorable or unfavorable traits within the breeding program and subsequently identify early generation material suitable as parents for crossing to exploit those favorable SNPs. MAB can be used to eliminate genotypes with unfavorable SNPs, thereby increasing selection efficiency. Reducing the number of genotypes with unfavorable characteristics at early generations in the program reduces required resources because the number of lines from each cross advanced to the next generation is reduced. Therefore, the use of breeding genotypes in association mapping can increase the rate of genetic improvement by increasing selection efficiency using both known and newly discovered regions associated with disease resistance in this specific study. Even better is the fact that those alleles are already present within the breeding population, which facilitates the development of commercial cultivars. Several limitations are inherent when using breeding populations for GWAS. New QTL for future introgression into the breeding program are not identified. Only QTL existing in the program can be identified, mapped, and exploited for MAB. The ability to identify QTL is limited by the diversity, both phenotypic and genotypic, of the breeding population. Rare alleles within the population may not be identified unless the MAF in the SNP data set was carefully chosen.

Historically, biparental populations and, later, diversity panels have been used for mapping. Shi et al. (2011) deviated from the use of biparental populations and used a breeding population consisting of 469 individuals for association mapping, with 75 SNP-based markers spread across the dry bean genome to identify QTL for CBB resistance. The population consisted of 91 cultivars and 116 advanced and 262 preliminary lines and included lines from both the Andean and Middle American gene pools. The population had a high LD; however, significant QTL within the population were still identified. Fifteen of 25 previously described QTL were confirmed, and eight new QTL were described. This initial evidence demonstrated that breeding populations can be used for association mapping to identify the QTL present in multiple genetic backgrounds. However, a marker density of six markers per chromosome severely limits the ability to identify candidate genes or new markers for MAB.

In the current study, breeding lines from various breeding stages and both gene pools within the NDSU Dry Bean Breeding Program were genotyped and evaluated for CBB resistance. The over 30,000 Andean SNPs and over 40,000 Middle American SNPs utilized in the current research are substantially more than the 75 SNP markers used by Shi et al. (2011), allowing the confirmation of six previously reported QTL, two new minor effect QTL, candidate gene selection, and marker selection for MAB. A smaller number of SNPs and reduced recombination found in the Andean SNP data set compared to the Middle American SNP data set was expected for two reasons. The Andean breeding population consisted only of beans from one race (Nueva Granada), and the genotypes are more closely related than the genotypes in the Middle American population belonging to two races. Additionally, the Andean SNP data set contained 139 individuals compared to the 713 individuals found within the Middle American data set. The combination of a smaller SNP data set and fewer genotypes did not hinder the identification of previously reported major effect QTL such as the one near the SAP6 region.

Field testing within the North Dakota State University dry bean breeding program consists of two stages (see footnote 1). Initially, many genotypes, generally at the F5 stage of inbreeding, are grown at a few locations over several years (preliminary yield trials). A subset of genotypes demonstrating good agronomic performance in preliminary yield trials was grown in advanced yield trials across multiple locations and years. Each trial is specific to a single dry bean market class (pinto, great northern, *etc*.). Phenotyping all lines in the NDSU breeding program provided a snapshot of the status of CBB resistance as well as the identification of adapted sources of resistance for further use within the breeding program. The results indicate that over 40% of genotypes within the program have CBB resistance at either the unifoliate or trifoliate stages, or both, and can be advanced and/or used as parents in subsequent crosses. A high frequency and number of resistant small red (92%; 47 genotypes) lines indicate that this market class is well positioned in regard to CBB resistance going forward. The frequency of CBB resistance in light red kidney (64%; 16 genotypes) is high, but the number of genotypes evaluated was relatively low. The frequency of CBB resistance for pinto (34%; 21 genotypes), great northern (25%; 13 genotypes), and dark red kidney (26%; 25 genotypes) beans is somewhat low, but a relatively high number of genotypes were screened, resulting in resistance being identified in numerous genotypes. The number of CBB-resistant genotypes identified is particularly low for black (six genotypes), pink (three genotypes), navy (two genotypes), and white kidney (six genotypes) beans, suggesting that crosses made with the CBB-resistant genotypes identified here should be increased, particularly with black and navy, which represent a moderate level of the market share in the region. Several VAX germplasm lines (Singh et al., 2001) have high levels of CBB resistance. These lines have been intensively used to introgress CBB resistance into elite parents during cultivar development. It is likely that the higher level of CBB resistance found in the small red market class is due to the frequent use of VAX-3, a small red-seeded line. Breeding efforts are improving the level of CBB resistance as evidenced by the significant difference between preliminary yield trial medians and advanced yield trial medians (unifoliate: 4.1 *vs.* 4.9, respectively, *p*-value < 0.0001; trifoliate: 4.2 *vs.* 4.7, respectively, *p*-value < 0.0001).

The GWAS identified SNPs associated with CBB resistance on six *P. vulgaris* chromosomes. One major region on Pv10 was confirmed as significantly associated with CBB resistance in both the Middle American and Andean breeding populations, explaining 10.8–16.9% in the Middle American population and 26.7–36.4% of the variation in the Andean population. In the Middle American population, the significant SNPs targeted a region (200 kb) centered at 41.78 Mb on Pv10 amidst a family of *LOX* genes. This region consisted of only 16 identifiable genes, of which 13 were *LOX*-1 orthologs. The LOX pathway is important in plant–pathogen interactions. Various compounds synthesized in the LOX pathway are signaling molecules, antimicrobials, and cytotoxic molecules (reviewed in Feussner and Wasternack, 2002). Two published examples (Croft et al., 1993; Ongena et al., 2004) associated the LOX pathway in *P. vulgaris* with plant–microbe interactions. Croft et al. (1993) investigated the accumulation of several LOX pathway-synthesized volatile lipids, *trans*-2hexenal and *cis*-3-hexanol, in *P. vulgaris* leaves after infection with *Pseudomonas syringae* pv. *phaseolicola*, the causal agent of halo blight in dry bean. No accumulation of the investigated volatile lipids was detected in buffer-inoculated leaves. In susceptible genotypes, less accumulation was detected when compared to the resistant genotypes. Both the volatile compounds had varying levels of antimicrobial activity, providing evidence of antimicrobial activity resulting from activation of the LOX pathway in *P. vulgaris*. Ongena et al. (2004) inoculated *P. vulgaris* plants with a non-pathogenic rhizobacteria and detected increases of linoleic and linolenic acids, suggesting the stimulation of the LOX pathway. Subsequent inoculation with fungal pathogens found decreased susceptibility associated with this prior exposure to non-pathogenic rhizobacteria. The results implicated the involvement of the LOX pathway in induced systemic resistance.

All the regions associated with CBB resistance in this study contained genes with a known link to disease reactions. The major region on Pv10 was unique in this study, as it primarily contained a cluster of orthologs for the same gene and is therefore a likely candidate gene. The other regions associated with CBB resistance have genes/orthologs known to be associated with disease resistance, including NB-AR domain-containing proteins, leucine-rich repeat receptor-like protein kinases, phosphatidylinositol-specific phospholipase C protein, pyridoxal phosphate phosphatase-related proteins, and cytochrome 450 proteins. Furthermore, six of the eight regions identified were near the physical location of previously described major and minor QTL. The finding of these genes within the associated region and the nearby localization with previously reported QTL supports the suitability of using breeding populations to map major and minor QTL already in the population and provides a starting point for future research to further delineate candidate genes. Other SNP falling just above the 0.1% cutoff may be indicative of additional minor QTL; however, they would contribute less than 5% of the phenotypic variation.

The identification of similar genomic regions associated with a single trait, in this study, CBB resistance, in both the Middle American and Andean populations was contrary to recent studies that have studied domestication, flooding, and root rot resistance traits. Schmutz et al. (2014) looked at domestication-related candidate genes and found very little overlap between candidate genes between the two gene pools. Soltani et al. (2017, 2018) examined flooding tolerance in both the Middle American and Andean gene pools. Very few associated genomic regions were similar between the two gene pools, indicating that different mechanisms are associated with flood tolerance. Oladzad et al. (2019b) identified resistance to *Rhizoctonia solani* in both gene pools, with no similar resistance regions identified. Similarly, Zitnick-Anderson et al. (2020) found no similarity in significant regions associated with resistance to *Fusarium solani* between the two gene pools. In contrast, the similarity of the associated locations of CBB resistance in this study suggests that a similar genetic factor or mechanism produces the phenotype in both the Andean and Middle American populations.

Genome-wide association studies produced several SNPs that may be targeted for MAB. Each of the peak SNPs identified in this study was evaluated for their utility in MAB by comparing the nucleotide state to the level of resistance found in the genotype. Selection for lines containing only the nucleotide associated with CBB resistance in the Andean population would remove two-thirds of the genotypes, of which more than half have some level of resistance in either or both developmental stages. This level of selection for only one trait would significantly increase the number of lines that would need to be genotyped to generate a large-enough pool for cultivar selection. The SNP nucleotide analysis in the Middle American population provided several targets for MAB. MAB based on SNP S07_28,772,508 would be advantageous in that it would primarily remove only CBB-susceptible genotypes and leave a large pool for further MAB and cultivar selection. Early removal of unacceptable phenotypes from the breeding program allows a more efficient use of program resources, even if only 14% reduction of the number of field-tested genotypes. As more genotypes enter the yield trials of the breeding program, their genotypes are being added to the data set to further delineate SNPs of interest. Two peak SNPs were located near SAP6 and SU91, two markers currently used for MAB. The peak SNP near SAP6, S10_41784824, was weakly correlated with SAP6 (correlation coefficient = 0.29), and SU91 was moderately correlated with S08_62399301 (correlation coefficient = 0.56).

The SNP data set generated in this study is being further exploited. Agronomic traits including seed yield and size, flowering date, and maturity date are undergoing association mapping to identify associated SNPs in order to confirm the previous findings from Agarwal (2014). Currently, a subset of the population is undergoing evaluations for resistance to rust (*Uromyces appendiculatus*), anthracnose (*Colletotrichum lindemuthianum*), and bean common mosaic necrotic virus to identify SNPs within the breeding population. Other traits of interest can be evaluated in these populations, including resistance to other pathogens and abiotic stresses. The genome-wide association studies provide valuable information in identifying the physical locations of resistance. Useful MAB SNPs identified for these other traits can be combined with the Pv07 SNP, S07_28,772,508, to generate a panel of markers used to reduce the number of genotypes with unacceptable phenotypes undergoing field testing.

## Conclusion

Dry bean breeding populations can be used for GWAS studies to identify previous linkage-mapped QTL, new genomic regions associated with traits of interest, candidate genes, and SNPs to target for future marker development. The CBB resistance test case using breeding populations in GWAS successfully identified genotypes with high levels of resistance and identified eight regions associated with resistance, including six previously discovered using biparental mapping and two new regions, one each on Pv11 and Pv10 in the Middle American population. The more diverse Middle American population led to the identification of several candidate genes for CBB disease resistance, including LOX orthologs as well as several SNPs to validate for MAB. Identification of haplotypes containing significant levels of CBB resistance and utilizing them during selection can provide a powerful strategy for dry bean improvement. Breeding population utilization for genetic studies allows the overlapping use of developed plant resources instead of generating usage-specific plant resources.

## Data Availability Statement

The datasets presented in this study can be found in online repositories. The names of the repository/repositories can be found below: https://hdl.handle.net/10365/31610, 2015 Snapshot.

## Author Contributions

KS, JO, PM, and JP conceived the original research plans, analyzed the data, and wrote the manuscript. KS, RL, and M performed the phenotyping experiments. KS generated the SNP data sets. AO performed the GEMMA analysis. All the authors read and approved the final manuscript.

## Conflict of Interest

The authors declare that the research was conducted in the absence of any commercial or financial relationships that could be construed as a potential conflict of interest.

## Footnotes

1 https://www.ag.ndsu.edu/plantsciences/research/dry-bean

2 http://hannonlab.cshl.edu/fastx_toolkit/

3 http://phytozome.jgi.doe.gov

4 http://broadinstitute.github.io/picard/

5 http://legumeinfo.org

6 https://phytozome.jgi.doe.gov/pz/portal.html
